# The Therapeutic Potential of Phages in Multi-Drug Resistance Infections and Future Directions

**DOI:** 10.3390/v18090937

**Published:** 2026-08-27

**Authors:** Shengting Zhang, Huili Tao, Sha Zhao, Yunlin Wei

**Affiliations:** 1 School of Ethnic Medicine, Yunnan Minzu University, Kunming 650500, China; 2 Faculty of Life Science and Technology, Kunming University of Science and Technology, Kunming 650500, China

**Keywords:** phage therapy, antimicrobial resistance, precision anti-infectives, phage–antibiotic synergy, regulatory science, personalized biologics

## Abstract

Phage therapy has been revisited as a biologically based strategy to tackle the escalating global crisis of multidrug-resistant (MDR) bacterial infections. Distinct from conventional antibiotics, bacteriophages target specific bacterial strains precisely, replicate locally at infection sites, penetrate bacterial biofilms, and exert synergistic effects with multiple antimicrobial agents. These inherent mechanistic advantages minimize collateral damage to the host’s commensal microbiota. However, existing regulatory frameworks—originally established for chemically synthesized, mass-produced drugs—fail to accommodate personalized, living biological phage products, leading to uncertain approval pathways and inconsistent manufacturing supervision. Clinical experience of phage therapy is predominantly derived from compassionate-use cases via multiple administration routes, including intravenous, inhaled, and topical delivery. This review systematically analyzes major challenges restricting clinical application, such as standardized production, quality control, pharmacokinetic characterization, rapid pathogen identification, and regulatory adaptation, as well as the limited performance of fixed phage cocktails against genetically heterogeneous bacterial populations. Current clinical practice demonstrates that phage therapy exhibits acceptable safety profiles across intravenous, inhaled, and topical administration routes, with promising therapeutic outcomes in otherwise untreatable MDR infections. Nevertheless, stable and reproducible clinical outcomes are hindered by multiple scientific and operational obstacles: the absence of unified standards for phage production and quality control, insufficient understanding of route-dependent pharmacokinetics, the imperative demand for rapid pathogen identification to enable precise phage matching, and the limited efficacy of fixed-cocktail regimens against genetically diverse clinical isolates. The successful integration of phage therapy into routine clinical practice relies on coordinated progress in diagnostic infrastructure construction, GMP-compliant phage repository establishment, international regulatory harmonization, and high-quality evidence generation through well-designed clinical trials. Rather than serving as a universal substitute for antibiotics, phage therapy is best implemented as a precision complementary component within comprehensive antimicrobial stewardship strategies.

## 1. Introduction

The discovery and deployment of antibiotics stand among the most transformative achievements in twentieth-century medicine, turning once-fatal bacterial infections into treatable conditions. Yet this cornerstone of modern healthcare is now crumbling under the relentless global spread of antimicrobial resistance. The World Health Organization has identified antimicrobial resistance (AMR) as one of the top ten threats to global public health, warning that without decisive action, drug-resistant infections could claim up to 10 million lives annually by 2050—a toll exceeding that of the 2008 financial crisis in economic impact alone [1]. At the heart of this crisis lies the alarming rise in multidrug-resistant (MDR), extensively drug-resistant (XDR), and pandrug-resistant (PDR) pathogens. Priority organisms such as carbapenem-resistant Enterobacterales (CRE), methicillin-resistant *Staphylococcus aureus* (MRSA), and carbapenem-resistant *Acinetobacter baumannii* (CRAB) are increasingly implicated in both nosocomial and community-acquired infections [2]. Their presence not only elevates mortality, prolongs hospital stays, and inflates healthcare expenditures but also jeopardizes the feasibility of routine medical interventions—from elective surgeries and organ transplantation to cancer chemotherapy—by undermining our capacity to prevent or manage life-threatening infections.

Compounding this threat is the stark inadequacy of the antibiotic development pipeline. Over the past three decades, the introduction of novel antibacterial agents has dwindled dramatically, and pharmaceutical investment in this area has waned due to formidable scientific hurdles and poor commercial incentives. While alternative strategies, including antimicrobial peptides, monoclonal antibodies, photodynamic therapy, and next-generation vaccines, continue to expand the range of options for preventing and treating infection, their clinical applicability may vary according to pathogen spectrum, delivery requirements, manufacturing considerations, and treatment setting. Rather than replacing established antimicrobial regimens, complementary approaches that can be readily integrated with current therapies may therefore offer additional value, particularly where they enhance antimicrobial efficacy or reduce dependence on conventional agents. Consequently, there is an urgent clinical need for innovative therapeutics that operate through distinct mechanisms, effectively bypass existing resistance determinants, and synergize with current antimicrobial regimens.

It is within this context that phage therapy—a century-old yet long-overlooked strategy—is experiencing a remarkable scientific and clinical renaissance. Phages, viruses that specifically infect and lyse bacteria, were first proposed as therapeutic agents by Félix d’Hérelle in 1917 and saw early use worldwide before the antibiotic era [3]. With the advent of penicillin and other broad-spectrum antibiotics, phage therapy faded from mainstream Western medicine, though it persisted in research and clinical practice in parts of Eastern Europe, notably Georgia and Poland [4,5]. Today, the resurgence of interest is driven overwhelmingly by the escalating AMR crisis. In cases where conventional options have been exhausted, phage therapy—administered under compassionate-use protocols—has successfully rescued critically ill patients, including a widely publicized case at the University of California, San Diego, involving a systemic infection with MDR A. baumannii. Such successes have catalyzed global attention and spurred renewed investment [6]. Moreover, advances in microbiome science, high-throughput genomics, and rapid screening technologies now enable systematic isolation, characterization, and banking of therapeutic phages, while synthetic biology offers unprecedented opportunities to rationally engineer phages for enhanced potency, expanded host range, and improved safety profiles [7].

This review provides a comprehensive and critical appraisal of the current state, challenges, and future trajectories of phage therapy for MDR bacterial infections. We begin by examining the fundamental biology of phages—their taxonomy, life cycles, and mechanisms of bacterial killing—and explore their potential for synergy with conventional antibiotics. We then synthesize evidence spanning preclinical models to completed and ongoing Phase I–III clinical trials, evaluating efficacy, safety, and the practical realities of different therapeutic paradigms. Key translational barriers are analyzed in depth, encompassing scientific uncertainties, manufacturing complexities, quality control standards, and regulatory incongruities. Looking ahead, we highlight emerging innovations: engineered phages equipped with CRISPR-Cas systems or biofilm-degrading enzymes, advanced delivery platforms, and rational combination strategies. Finally, we propose a roadmap for transitioning phage therapy from an emergency, patient-specific intervention toward a standardized, accessible, and sustainable component of the global antimicrobial arsenal—offering actionable insights for researchers, clinicians, regulators, and policymakers alike. The present article was designed as a critical narrative review rather than a systematic review, with the aim of integrating mechanistic, preclinical, clinical, and translational evidence relevant to the application of phage therapy against multidrug-resistant bacterial infections. In contrast to previous reviews that have primarily summarized phage biology, therapeutic applications, or individual translational barriers, the present review adopts an integrative and critical perspective linking phage–host biology with the factors that ultimately determine clinical performance. Particular emphasis is placed on distinguishing receptor-mediated adsorption from productive host range, considering the contribution of intracellular bacterial defense systems, phage resistance, propagation-host effects, and phage–antibiotic interactions, and examining how these biological variables intersect with manufacturing quality, formulation, route of administration, pharmacokinetics, host immune responses, and regulatory requirements. In parallel, the clinical literature is evaluated according to the strength of the underlying evidence, with compassionate-use reports, uncontrolled studies, and early-phase trials distinguished from controlled clinical investigations. Through this framework, the review seeks not merely to catalogue advances in phage therapy, but to identify the biological and translational conditions under which phage treatment is most likely to provide a meaningful advantage over optimized conventional therapy.

## 2. Methods

### 2.1. Phage Taxonomy and Life Cycle Strategies

Phages—viruses that exclusively infect bacteria—represent the most abundant and genetically diverse biological entities on Earth [8]. They inhabit virtually every ecosystem where bacteria thrive, including soil, aquatic environments, and the human microbiome [9]. Phages have re-emerged as promising therapeutic agents against multidrug-resistant (MDR) pathogens because of their high host specificity and potent lytic activity. Accordingly, understanding their taxonomic diversity and life cycle strategies is important for the rational selection, design, and safe application of phage-based therapeutics [10,11].

According to the current taxonomic framework established by the International Committee on Taxonomy of Viruses (ICTV), tailed double-stranded DNA (dsDNA) bacteriophages are classified within the class Caudoviricetes [12,13,14]. The historically recognized morphology-based families Myoviridae, Siphoviridae, and Podoviridae, together with the order Caudovirales, have been abolished because these groups were shown to be polyphyletic and did not adequately reflect the evolutionary relationships among tailed phages [12]. Nevertheless, the terms myovirus, siphovirus, and podovirus may still be used as non-taxonomic morphological descriptors for phages possessing contractile tails, long non-contractile tails, and short tails, respectively [12]. Under the current ICTV framework, members of Caudoviricetes are instead assigned to genome-defined orders, families, genera, and species based primarily on genomic relatedness and phylogenetic relationships [12,13,14]. This genome-based framework provides a more evolutionarily coherent approach for the classification and systematic organization of tailed bacteriophages [12,13,14]. While dsDNA phages dominate clinical pipelines, other genomic architectures—including single-stranded DNA (ssDNA), double-stranded RNA (dsRNA), and single-stranded RNA (ssRNA)—also occur in nature [14]. However, these phages are rarely pursued for therapeutic use because of factors such as limited host ranges, complex replication mechanisms, or insufficient characterization. Phage host specificity is determined by multiple sequential barriers to productive infection. Initial host recognition is mediated largely by interactions between viral receptor-binding proteins (RBPs), typically located on tail fibers or spikes, and bacterial surface structures such as lipopolysaccharides, outer membrane proteins, pili, flagella, or teichoic acids [15,16,17,18,19]. These receptor–ligand interactions can be highly specific and are often described as a “lock-and-key” mechanism, thereby restricting adsorption to particular bacterial species or strains. However, successful adsorption alone does not necessarily result in productive infection. Following genome entry, phage replication may be further restricted by intracellular bacterial defense systems, including restriction–modification systems, CRISPR-Cas immunity, abortive infection mechanisms, and other antiphage defense pathways. Consequently, phage host range reflects the combined effects of receptor compatibility and the ability of the phage to overcome intracellular barriers. At the structural level, RBPs contribute to the initial recognition step through shape complementarity and non-covalent interactions, including hydrogen bonding and electrostatic forces, with specific bacterial surface ligands. Binding often induces conformational changes in the tail apparatus that trigger genome ejection; even single-residue substitutions in RBPs or their cognate receptors can abrogate infection, accounting for the narrow host range typical of therapeutic phages.

Phage life cycles are commonly described in terms of two canonical strategies, lytic and lysogenic, although in practice phage replication behaviors can occur along a continuum rather than fitting strictly into either category. At the lytic end of this spectrum, phages initiate replication soon after infection: following adsorption and genome injection, they redirect host transcriptional and translational machinery toward the production of viral components, ultimately causing bacterial cell lysis and the release of progeny virions, often within 20–60 min [20]. Because of their rapid bactericidal activity and lack of stable prophage formation, obligately lytic phages are generally preferred for therapeutic applications. At the lysogenic end, temperate phages can integrate their genomes into the bacterial chromosome, or otherwise persist within the host as prophages, and replicate with the bacterial cell until environmental or physiological cues promote induction into a lytic program [21]. Importantly, these categories represent useful conceptual endpoints, as phages may display intermediate or context-dependent replication strategies influenced by both viral and host factors. Although lysogeny plays a pivotal role in bacterial evolution—sometimes conferring fitness advantages such as toxin production or antibiotic resistance—it poses significant safety concerns in clinical settings due to the risk of horizontal gene transfer. Consequently, phages selected for therapeutic use undergo stringent genomic screening to exclude integrase genes, virulence factors, and antimicrobial resistance determinants, ensuring they are obligately lytic and devoid of undesirable genetic cargo. Nevertheless, successful infection by even obligately lytic phages is contested by a sophisticated array of bacterial anti-phage defenses. Bacteria deploy multilayered immunity: surface modifications (e.g., capsule masking or receptor loss) block adsorption; restriction–modification (R–M) systems cleave unmethylated phage DNA; abortive infection modules induce altruistic cell death to limit viral spread; and CRISPR-Cas provides adaptive, sequence-specific immunity through targeted DNA degradation. In turn, phages counter these barriers by methylating their genomes to evade R–M recognition, mutating protospacers or PAM sites to escape CRISPR surveillance, encoding anti-restriction proteins, or producing anti-CRISPR proteins that directly inhibit Cas nuclease activity [22]. This dynamic co-evolutionary arms race not only drives genetic diversification but also has direct implications for therapy—underscoring the need to select phages that lack anti-CRISPR genes (to avoid unintended immunosuppression) and to demonstrate robust infectivity against clinical isolates despite active host defenses.

### 2.2. Antimicrobial Mechanisms of Phages: From Host Recognition to Bacterial Lysis

Having overcome bacterial defenses, successful phages proceed through a tightly orchestrated, multi-step infection and lysis cascade. Unlike conventional antimicrobials that rely on biochemical inhibition or membrane disruption, phages eliminate bacterial pathogens through a tightly orchestrated, multi-step infection and lysis cascade. Following successful adsorption, the phage delivers its genetic material into the bacterial cell. The mechanism of genome entry varies among phages and is coordinated with structural changes in the virion during attachment [18,19]. Upon successful genome delivery, the phage genetic material enters the bacterial cytoplasm, where replication depends on the ability of the phage to utilize or redirect the host’s transcriptional and translational machinery [23]. Early viral genes suppress native bacterial metabolism, while middle and late genes direct the replication of phage nucleic acids and the synthesis of structural components, culminating in the assembly of hundreds of progeny virions within a single replication cycle.

The terminal phase of this lytic program hinges on the coordinated action of two evolutionarily conserved proteins: holins and endolysins. Holins accumulate in the inner membrane and, at a genetically programmed time, form micropores that allow endolysins—peptidoglycan-degrading enzymes—to translocate from the cytoplasm into the periplasmic space [24]. There, endolysins cleave critical bonds within the peptidoglycan layer, either glycosidic linkages or peptide cross-bridges, leading to catastrophic loss of cell wall integrity, osmotic imbalance, and rapid bacterial lysis. In Gram-negative bacteria, some phages further encode auxiliary lysis proteins known as spanins, which disrupt the outer membrane by fusing it with the compromised inner membrane, thereby ensuring complete disintegration of the cell envelope [25]. Importantly, resistance to phage infection—often arising from mutations in surface receptors—typically imposes a fitness cost on the bacterium, such as reduced virulence or impaired growth, making durable resistance less likely to emerge compared to antibiotic pressure.

Beyond planktonic killing, phages exhibit a distinctive capacity to combat biofilm-associated infections, a major clinical challenge in chronic wounds, indwelling medical devices, and cystic fibrosis airways [26]. Biofilms, encased in a self-produced extracellular polymeric matrix, are notoriously refractory to antibiotics due to limited penetration and altered metabolic states of embedded cells. Certain phages, however, can actively penetrate this barrier and propagate locally through a “diffuse-and-reinfect” cycle: lysis of surface-layer bacteria releases new virions that infect deeper layers, progressively eroding the biofilm architecture [27]. Moreover, many therapeutic phages carry genes encoding depolymerases—enzymes that specifically degrade key matrix components such as alginate, cellulose, or exopolysaccharides [28]. By dismantling the structural scaffold of the biofilm, these enzymes not only enhance phage access but also sensitize embedded bacteria to co-administered antimicrobials. This dual functionality positions phages as uniquely suited agents for tackling resilient, biofilm-mediated MDR infections.

### 2.3. Synergistic Dynamics Between Phages and Antibiotics

The notion that phages and antibiotics must operate in isolation has been decisively overturned by a growing body of evidence demonstrating potent synergistic interactions between these two antimicrobial modalities [29]. Far from being mutually exclusive, their combination often yields enhanced bacterial killing, delayed resistance emergence, and even resensitization of resistant strains to otherwise ineffective drugs—a phenomenon collectively termed phage–antibiotic synergy (PAS) [30]. This synergy arises not from simple additive effects but from complex, bidirectional biological crosstalk that reshapes both bacterial physiology and phage replication dynamics.

One well-documented mechanism involves subinhibitory concentrations of certain antibiotics—particularly those targeting cell wall synthesis (e.g., β-lactams) or protein translation (e.g., aminoglycosides)—which can inadvertently enhance phage propagation. For instance, β-lactams induce the bacterial SOS response and upregulate membrane porins or phage receptors, thereby increasing phage adsorption efficiency [31]. Critically, some antibiotics also suppress bacterial anti-phage defenses—for example, by downregulating CRISPR-Cas expression or impairing restriction–modification activity—thereby rendering cells more permissive to phage infection. Simultaneously, antibiotic-induced metabolic stress may accelerate phage DNA replication or delay host lysis inhibition, leading to higher burst sizes [32]. Conversely, phage infection can alter bacterial membrane permeability or downregulate efflux pumps, thereby potentiating intracellular antibiotic accumulation. In *Pseudomonas aeruginosa*, for example, phage-mediated suppression of the MexAB-OprM efflux system has been shown to increase susceptibility to fluoroquinolones and enhance the antibacterial activity of tetracyclines, despite the intrinsic resistance of *P. aeruginosa* to this antibiotic class [33].

Critically, PAS can also suppress the evolution of resistance. In vitro evolution experiments reveal that while bacteria readily develop resistance to either phages or antibiotics when used alone, dual exposure dramatically reduces the frequency of double-resistant mutants [34]. This is partly because resistance mutations often incur conflicting fitness costs: a mutation that alters a surface receptor to evade phage adsorption may simultaneously increase antibiotic uptake, rendering the cell more vulnerable. Moreover, some phages have been engineered to deliver CRISPR-Cas systems targeting antibiotic resistance genes, effectively “re-sensitizing” MDR pathogens during infection—a strategy that merges genetic editing with antimicrobial therapy [35].

These principles have begun to translate into clinical practice. Compassionate-use cases increasingly employ phage–antibiotic combinations, with notable success in treating prosthetic joint infections, osteomyelitis, and chronic lung infections in cystic fibrosis patients. In this specific population—individuals with cystic fibrosis receiving repeated nebulized phage therapy for chronic respiratory infections—recent data indicate that treatment is generally well tolerated, with no acute hypersensitivity reactions observed; however, neutralizing antibodies against the administered phages were detected in a subset of patients, suggesting a potential immune-mediated limitation to sustained efficacy [36]. Beyond these indications, emerging evidence suggests that phage–antibiotic synergy may also extend to highly recalcitrant pathogens, including drug-resistant mycobacteria. In a compassionate-use series of 20 patients with severe, drug-resistant nontuberculous mycobacterial infections, personalized intravenous phage therapy administered alongside antibiotics led to clinical improvement in 55% of cases and was well tolerated [37]. Supporting this trend, a five-year experience from the Israeli Phage Therapy Center reported remission or recovery in 77.8% of patients receiving personalized phage therapy alongside antibiotics for otherwise refractory infections [38]. In a retrospective analysis of 12 patients receiving individualized phage therapy, in vitro synergy between phages and antibiotics was observed in the majority of cases. Clinical improvement was documented in 58% of patients, and when combined with microbiological eradication of the target pathogen, the overall favorable response rate rose to 66% [11]. A recent compassionate-use study involving 16 patients with refractory *Pseudomonas aeruginosa* infections—predominantly osteoarticular or associated with indwelling medical devices—reported favorable clinical outcomes in 13 of 15 evaluable cases (86.6%) following combination therapy with the lytic phage PASA16 and antibiotics, with only mild adverse effects observed [39]. Because compassionate-use phage therapy has been reported across a broad and heterogeneous range of pathogens, infection syndromes, and treatment protocols, Table 1 is intended to provide representative rather than exhaustive examples of the available clinical experience. Studies were selected from the literature discussed in this review on the basis of their reporting of human clinical outcomes, relevance to difficult-to-treat or antimicrobial-resistant infections, and ability to illustrate different clinical contexts, pathogens, and routes of phage administration. Where available, larger case series and institutional experiences were prioritized over individual case reports. Given the predominantly uncontrolled and heterogeneous nature of these studies, their findings should be interpreted as descriptive evidence of feasibility, safety, and potential clinical activity rather than as definitive estimates of treatment efficacy.

Nevertheless, synergy is not universal—it is highly context-dependent, varying by phage–antibiotic pair, bacterial species, and infection microenvironment. Indiscriminate pairing can even yield antagonism, as seen when bacteriostatic antibiotics (e.g., tetracyclines) suppress bacterial metabolism to levels incompatible with phage replication. Thus, rational design of combination regimens demands systematic screening and mechanistic understanding. Emerging platforms integrating time-kill kinetics, transcriptomics, and machine learning are now being deployed to predict optimal pairings, paving the way for precision phage–antibiotic co-therapy tailored to individual pathogens and resistance profiles.

## 3. Results

### 3.1. Key Findings from Preclinical Research Models

Preclinical studies serve as the cornerstone for translating phage therapy from bench to bedside, providing essential insights into the efficacy, safety, and mechanisms of action against MDR pathogens. These investigations typically encompass in vitro assays and animal models, offering a comprehensive evaluation of phage performance under controlled conditions.

In vitro experiments have demonstrated the potent antibacterial activity of various phages against MDR pathogens. For instance, a recently characterized lytic phage, SPB, exhibited robust activity against both planktonic and biofilm-associated methicillin-resistant *S. aureus* (MRSA), significantly reducing biofilm biomass in vitro [40]. Notably, genomic analysis revealed that SPB encodes a putative depolymerase, implicating enzymatic degradation as a key mechanism underlying its anti-biofilm efficacy. Expanding this approach to other high-priority Gram-negative pathogens, a novel phage designated ΦAb1656-2 was shown to effectively lyse MDR *Acinetobacter baumannii* strains, while its purified endolysin, LysAb1656-2, displayed even more potent and rapid bactericidal activity—highlighting the therapeutic potential of phage-derived enzymes as standalone agents or adjuncts to conventional therapy [41]. This strategy has now been further advanced through protein engineering: the engineered endolysin derived from phage ΦEcSw demonstrated enhanced antibacterial activity against an MDR *Escherichia coli* strain, achieving rapid cell lysis without requiring additional outer membrane disruptors—a significant step toward overcoming the inherent resistance of Gram-negative bacteria to exogenous lytic enzymes [42]. Together, these findings underscore that both whole phages and their engineered enzymatic derivatives—such as endolysins and depolymerases—can disrupt resilient bacterial structures, including biofilms, and offer versatile tools for targeting chronic and drug-resistant infections.

Animal infection models provide critical validation of phage therapy’s potential in vivo.

For example, the lytic phage PaeP_Ls, isolated against a MDR *Pseudomonas aeruginosa* clinical strain, demonstrated not only potent in vitro antibacterial and anti-biofilm activity but also significant therapeutic efficacy in a murine sepsis model: a single intraperitoneal administration markedly improved survival, reduced bacterial loads in the spleen and liver, and attenuated systemic inflammation [43]. In a murine model of ventilator-associated pneumonia caused by MRSA, combined nebulized and intravenous phage administration achieved superior therapeutic outcomes compared to monotherapy, with a remarkable 91% survival rate [44]. In another study, a single dose of a specific phage significantly improved survival in mice with sepsis or meningitis caused by a multidrug-resistant, ESBL-producing *E. coli* ST131 strain—one of the most common and dangerous drug-resistant clones worldwide [45]. Furthermore, the safety profile of phage therapy was rigorously assessed, revealing minimal adverse effects and no signs of toxicity, thus reinforcing its potential as a viable therapeutic option [46]. Overall, preclinical research has laid a solid foundation for advancing phage therapy into clinical trials, demonstrating its promise as a novel approach to combatting MDR infections.

### 3.2. Clinical Evidence for MDR Infections

The clinical evidence for phage therapy against MDR bacterial infections has emerged primarily through two complementary pathways: expanded access (compassionate use) programs and formal clinical trials, both of which reflect the tension between personalized intervention and standardized drug development. To date, multiple peer-reviewed compassionate-use cases of phage therapy have been reported. These include the successful treatment of a patient with chronic obstructive pulmonary disease (COPD) suffering from life-threatening ventilator-associated pneumonia due to carbapenem-resistant *Acinetobacter baumannii*, who showed marked clinical improvement after receiving a personalized phage cocktail via both nebulized and intravenous routes, with no treatment-related adverse effects [47]. In a particularly complex pediatric case, an infant with a dual pulmonary infection caused by carbapenem-resistant A. baumannii and *Klebsiella pneumoniae*—unresponsive to all available antibiotics—achieved clinical stabilization and microbiological clearance of *A. baumannii* following personalized aerosolized phage therapy, highlighting the potential of inhaled phages in vulnerable populations with polymicrobial MDR infections [48]. This clinical approach is further supported by preclinical proof-of-concept studies demonstrating that precisely tailored phage cocktails can achieve rapid eradication of carbapenem-resistant *A. baumannii* while simultaneously imposing evolutionary trade-offs on resistant mutants—such as impaired growth or reduced virulence—thereby constraining their long-term survival and spread [49]. Another well-documented compassionate-use case involves the successful resolution of a chronic MRSA prosthetic knee infection following a single intra-articular administration of a tailored phage cocktail, enabling prosthesis retention and sustained clinical cure without serious adverse effects [50]. However, these successes also highlight critical challenges in translating phage therapy into routine clinical practice. For instance, host factors such as plasma proteins can significantly impair phage infectivity—studies have shown that human plasma may reduce the infection efficiency of certain phages targeting *Staphylococcus aureus* by up to 98%, potentially due to binding of phage receptors on bacterial surfaces by serum proteins. This suggests that route of administration and dosing must be carefully optimized, particularly in systemic infections like bacteremia or prosthetic joint infections, where phages are exposed to high levels of plasma components [51].

Clinical trials have sought to systematize these observations. For example, a double-blind, placebo-controlled Phase I trial demonstrated that intranasal administration of a phage cocktail in patients with *Staphylococcus aureus*-positive chronic rhinosinusitis was safe and well-tolerated, with no serious adverse events reported [52]. In a prospective clinical study of 47 patients with chronic nonhealing wounds harboring MDR bacterial infections, topical phage therapy resulted in significant reduction or complete eradication of pathogenic bacteria in the majority of cases, leading to resolution of local infection signs—such as purulent discharge and inflammation—and enabling subsequent wound healing [53]. In a prospective phase I/II trial of intravenous phage therapy combined with antibiotics for severe *S. aureus* infections—including endocarditis, osteomyelitis, and pneumonia—17 of 20 patients achieved clinical cure or significant improvement, with high rates of microbiological clearance, underscoring the therapeutic potential of systemic phage treatment in life-threatening, difficult-to-treat infections [54]. This momentum has now extended beyond individual trials into structured clinical implementation: in 2022, the PHAGEinLYON Clinic at Hospices Civils de Lyon established a multidisciplinary pathway that evaluated 168 patient requests and delivered personalized phage therapy to 72 individuals with otherwise untreatable MDR infections, most of whom experienced meaningful clinical improvement, demonstrating the feasibility of integrating phage therapy into routine hospital care [55]. Collectively, the clinical evidence base reveals a maturing field: while early compassionate use pioneered individualized, isolate-matched regimens—often employing custom cocktails or single phages—modern trials are formalizing these principles within regulated frameworks. The choice among monophage therapy, tailored mixtures, pre-formulated cocktails, or combination regimens is no longer ad hoc but is being empirically refined based on infection site, pathogen diversity, and pharmacokinetic constraints. As regulatory agencies develop adaptive pathways for personalized biologics, phage therapy is transitioning from a last-resort intervention toward a structured, evidence-informed component of the antimicrobial arsenal against MDR infections (Table 2).

## 4. Current Challenges and Translational Barriers

The clinical integration of phage therapy remains constrained by a complex interplay of scientific, regulatory, and microbiological challenges that hinder reproducibility, scalability, and global adoption. One of the most fundamental hurdles lies in quality control and standardization: unlike conventional antibiotics, phage preparations are biological entities whose efficacy depends critically on titer, purity, stability, and formulation consistency [57]. The absence of universally accepted standards for phage characterization leads to substantial variability across studies and institutions, complicating cross-trial comparisons. Furthermore, phage formulations vary widely in physical form—liquid suspensions, lyophilized powders, or topical gels—and their stability under ambient conditions remains inconsistent, posing logistical challenges for storage and distribution, particularly in low-resource settings.

Compounding these issues is the limited understanding of pharmacokinetics and pharmacodynamics (PK/PD). Unlike small-molecule drugs, phages replicate at the site of infection, leading to self-amplifying dynamics that defy traditional PK models. Their clearance rates, tissue penetration, and interaction with host immune responses are poorly characterized, particularly across diverse routes of administration [58]. For instance, intravenous delivery may result in rapid hepatic sequestration, while nebulized phages face degradation in the airways or mucociliary clearance. Moreover, the diversity of administration routes further complicates therapeutic design: phages can be delivered locally (e.g., topical for wounds, intra-articular for joint infections), via inhalation for pulmonary infections, or systemically for disseminated disease [59]. Each route presents distinct bioavailability and exposure challenges, yet optimal dosing regimens remain largely empirical, driven more by case experience than evidence-based principles.

**Figure 1:**
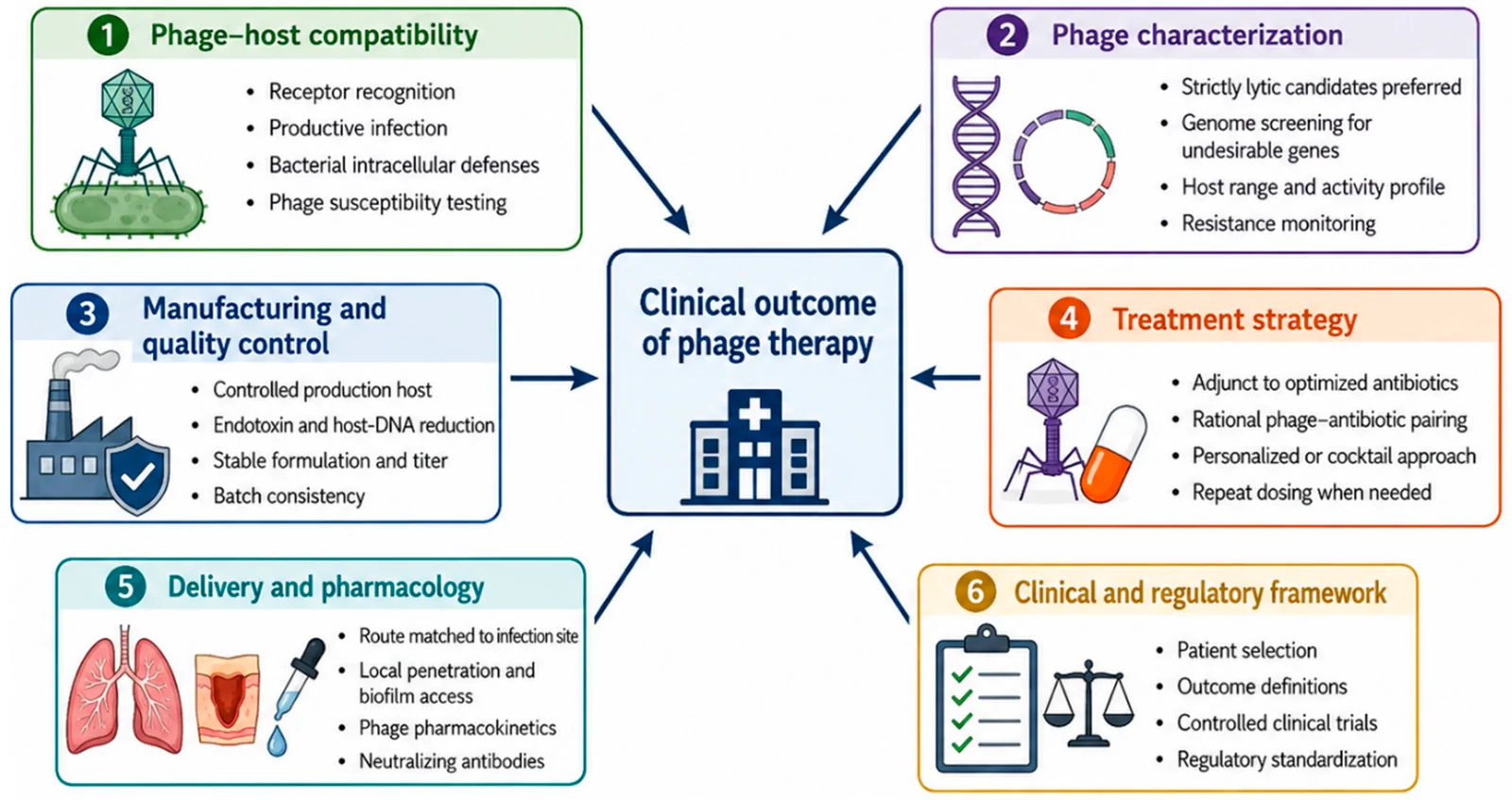
Key determinants of successful phage therapy against multidrug-resistant bacterial infections.

Regulatory frameworks represent another significant barrier. Most current systems are designed for mass-produced, standardized pharmaceuticals and struggle to accommodate personalized phage therapies tailored to individual patient isolates. In addition to genome-encoded determinants of host specificity, the bacterial strain used for phage propagation may, in some phage–host systems, influence the phenotypic properties of the resulting phage preparation. For example, propagation-host-dependent restriction–modification patterns have been shown to alter phage DNA methylation and host range in a *Staphylococcus aureus* phage system [60]. However, the prevalence and clinical significance of such effects across different phage–bacterium systems remain uncertain. These observations emphasize the importance of appropriate manufacturing-host selection and standardized production and quality-control procedures for therapeutic phages [61]. Emerging clinical frameworks, such as the POSTSTAMP study for treatment-refractory nontuberculous mycobacterial pulmonary disease in people with cystic fibrosis, are also contributing to more structured approaches for the prospective clinical evaluation of phage therapy [56,62]. Similarly, Japan’s Pharmaceuticals and Medical Devices Agency (PMDA) has begun issuing guidance on phage therapy, reflecting growing regulatory engagement [61]. Nevertheless, divergent national approaches continue to hinder international collaboration and scalable commercial development. In addition, at the microbial level, the morphology of bacteria and the heterogeneity of bacterial populations can also affect the effectiveness of phage therapy, as demonstrated by pre-clinical investigations using broad-host-range Myoviridae phages against multidrug-resistant bacterial isolates [63]. Figure 1 illustrates key determinants of successful phage therapy against MDR bacterial infections.

## 5. Discussion

**Figure 2:**
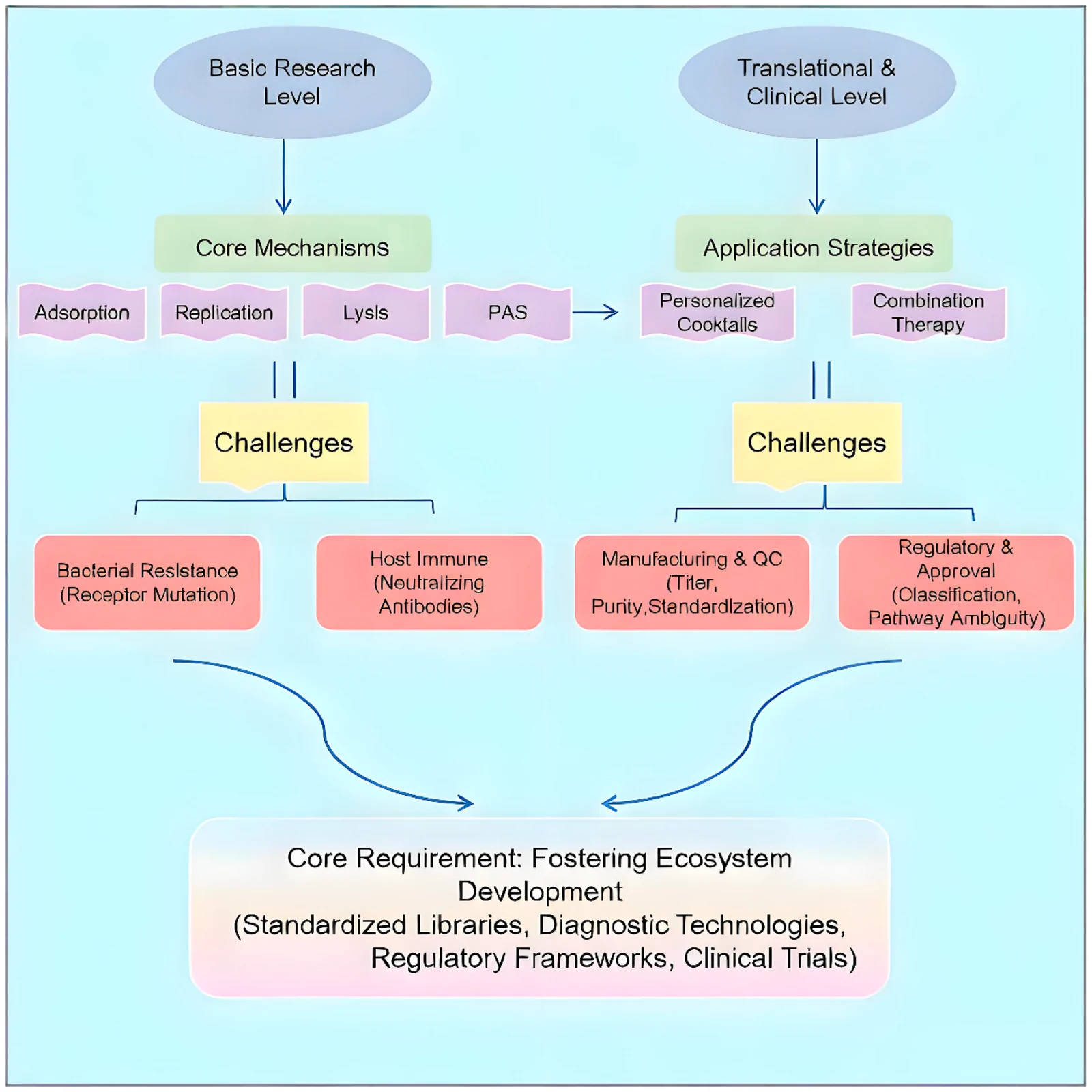
Conceptual framework for the development and translation of phage therapy, highlighting core mechanisms, clinical strategies, translational challenges, and ecosystem requirements. PAS, phage–antibiotic synergy; QC, quality control.

Phage therapy is gaining renewed attention not because of historical precedent, but because its biological properties address specific gaps in current anti-infective strategies—particularly for infections caused by MDR bacteria that no longer respond to available antibiotics. Phages kill target bacteria with high specificity, replicate at the site of active infection, penetrate biofilms, and can enhance the activity of certain antibiotics through synergistic interactions [27]. These features offer theoretical advantages over broad-spectrum agents that disrupt the microbiome and accelerate resistance selection. At the same time, the same specificity that enables precision also limits broad applicability, as effective treatment requires matching phages to the patient’s infecting strain—a process that depends on timely diagnostics and access to diverse phage collections. A conceptual framework summarizing these key mechanisms, along with the translational strategies, persistent challenges, and ecosystem requirements for advancing phage therapy, is presented in Figure 2.

Modern phage therapy differs substantially from the largely empirical approaches employed during the early 20th century. Contemporary therapeutic development generally prioritizes well-characterized lytic phages with appropriate antibacterial activity and host specificity, supported by phenotypic susceptibility testing and detailed genomic characterization [57]. Therapeutic phage preparations are also subject to increasingly stringent manufacturing and quality-control requirements, including control of bacterial contaminants, endotoxins, residual host-cell DNA, and other process-related impurities [61]. In parallel, genomic screening is used to assess phage identity and to exclude undesirable genetic determinants, particularly genes associated with bacterial virulence, lysogeny, or the potential mobilization of antimicrobial-resistance determinants [11,61]. These practices reflect major advances in genomics, molecular microbiology, manufacturing control, and regulatory science that were unavailable during the early development of phage therapy [57,61]. Consequently, contemporary phage therapy is more appropriately regarded as a rationally characterized and increasingly personalized biological therapeutic strategy rather than simply a revival of early empirical phage use [57,58].

The clinical evidence remains preliminary. Most data come from compassionate-use cases, which, while ethically necessary and occasionally life-saving, lack controls, standardized dosing, and long-term follow-up. Early-phase trials have consistently shown that phage administration—by intravenous, inhaled, or topical routes—is well tolerated, but efficacy signals are modest and variable. The Phagoburn trial highlighted a key operational challenge: fixed phage cocktails, though easier to manufacture and regulate, may fail when confronted with genetically diverse clinical isolates or suboptimal storage conditions [64]. This experience suggests that successful application often depends on real-time matching rather than pre-formulated products.

A major unresolved issue is the lack of pharmacokinetic and pharmacodynamic data. Unlike small-molecule antibiotics, phages multiply in the presence of susceptible hosts, leading to dynamic concentration changes that are difficult to model. Their distribution varies by route—intravenous doses are rapidly cleared by the reticuloendothelial system, while inhaled phages face enzymatic degradation and mucociliary clearance. Without reliable models to guide dosing, regimens remain largely based on anecdotal experience.

Future progress will depend on integrating phage therapy into coordinated care pathways. Rapid pathogen identification, susceptibility-guided phage selection, and access to GMP-compliant phage banks are prerequisites for consistent outcomes. Emerging tools—such as engineered phages with expanded host ranges or CRISPR-Cas systems targeting resistance genes—may enhance reliability, but their clinical utility remains unproven [65]. Equally important is regulatory evolution: agencies must develop flexible frameworks that distinguish between emergency personalized use, standardized cocktails for defined indications, and genetically modified products, each with appropriate safety and quality requirements.

International collaboration is essential. Harmonized standards for phage characterization, shared repositories, and multicenter trials would accelerate knowledge generation and avoid redundant efforts. Until such infrastructure exists, phage therapy will remain a promising but inconsistently applied intervention.

## 6. Conclusions

Current evidence supports phage therapy as a promising but highly context-dependent strategy for selected multidrug-resistant bacterial infections, rather than as a broadly interchangeable substitute for antibiotics. Across the preclinical and clinical literature reviewed here, therapeutic success appears to depend on several interconnected factors, including phage–host compatibility, bacterial intracellular defense mechanisms, infection site, route of administration, phage pharmacokinetics, host immune responses, and the concurrent use of antibiotics. Importantly, receptor recognition alone does not define therapeutic host range, and favorable in vitro activity cannot be assumed to translate directly into clinical efficacy. The available clinical evidence remains dominated by compassionate-use reports, small case series, and early-phase studies, many of which involve individualized phage preparations administered together with optimized antibiotic therapy. These studies provide important evidence of feasibility and generally acceptable tolerability but offer only limited ability to determine independent phage efficacy. Controlled trials, including PhagoBurn, further demonstrate that therapeutic performance can be strongly influenced by practical factors such as phage titre, formulation stability, dosing, and delivery. Thus, the principal challenge is no longer simply identifying bactericidal phages but developing reproducible treatment systems that preserve phage activity from selection and manufacturing through administration at the site of infection. A central synthesis emerging from this review is that the future clinical value of phage therapy is likely to lie in precision, combination-based treatment rather than universal broad-spectrum application. Rapid phage susceptibility testing, careful genomic and phenotypic characterization, rational phage–antibiotic pairing, standardized manufacturing, and monitoring of bacterial resistance and host immune responses will be critical to patient selection and therapeutic optimization. At the same time, harmonized regulatory standards and adequately powered controlled trials are required to distinguish true therapeutic benefit from outcomes attributable to concomitant antibiotics, surgery, or natural clinical variation. Accordingly, phage therapy should currently be regarded as an emerging precision anti-infective platform with particular potential for carefully selected, difficult-to-treat infections. Its transition from individualized compassionate use to routine clinical practice will depend less on demonstrating bactericidal activity alone and more on establishing when, how, and in which patients’ phages provide a measurable advantage over optimized standard care.

## Figures and Tables

**Table 1:** Representative Clinical Evidence from Compassionate-Use and Observational Studies of Phage Therapy Frequently Administered in Combination with Antibiotics.

Study Population and Infection Type	Study Design	Key Clinical Findings and Outcomes	References
Cystic fibrosis patients with chronic respiratory infections	Compassionate use; repeated nebulized phage therapy	Treatment was well-tolerated (no acute hypersensitivity reactions observed). Limitation: Neutralizing antibodies against the administered phages were detected in a subset of patients, suggesting a potential constraint on sustained efficacy.	[36]
Severe, drug-resistant nontuberculous mycobacterial infections	Compassionate-use series; personalized intravenous phage therapy + antibiotics	Clinical improvement rate: 55% (11/20) of cases. The therapy was reported to be well-tolerated.	[37]
Refractory infections (broad spectrum)	Five-year experience summary from a single center; personalized phage therapy + antibiotics	Remission or recovery rate: 77.8% of patients. Supports the efficacy trend of this therapy for difficult-to-treat infections.	[38]
Refractory infections	Retrospective analysis; individualized phage therapy	1. In vitro synergy: Observed in the majority of phage-antibiotic combinations. 2. Clinical improvement rate: 58% (7/12) of patients. 3. Overall favorable response rate (clinical improvement + microbiological eradication): 66%.	[11]
Refractory *Pseudomonas aeruginosa* infections (osteomyelitis/device-related)	Compassionate-use study; lytic phage PASA16 + antibiotics	Favorable clinical outcome: 86.6% (13/15) of evaluable cases. Only mild adverse effects were reported.	[39]

Note: The studies presented are representative examples and do not constitute an exhaustive or systematic review of all compassionate-use phage therapy cases. Differences in patient selection, phage composition, dosing, route of administration, concomitant antimicrobial therapy, and outcome assessment limit direct comparisons across studies.

**Table 2:** Representative preclinical and clinical evidence for bacteriophage-based approaches against drug-resistant bacterial infections.

Evidence Category	Pathogen/Infection Model	Intervention and Study Design	Main Findings	Critical Interpretation/Limitations	Ref.
Preclinical: in vitro	Methicillin-resistant *Staphylococcus aureus* (MRSA)	Lytic phage SPB; planktonic and biofilm assays	Phage SPB demonstrated broad lytic activity against the tested MRSA isolates and inhibited planktonic growth and biofilm formation.	Provides evidence of in vitro antibacterial and antibiofilm activity; however, in vitro susceptibility does not establish pharmacological activity or therapeutic efficacy in vivo.	[40]
	Multidrug-resistant *Acinetobacter baumannii*	Phage ΦAb1656-2 and phage-derived endolysin LysAb1656-2	The purified endolysin showed substantial antibacterial activity against MDR *A. baumannii* and, under the conditions examined, greater activity than the parental phage.	Supports the potential of phage-derived enzymes as antibacterial agents, but enzyme activity in vitro should not be extrapolated directly to therapeutic efficacy because stability, delivery, and pharmacokinetics remain important barriers.	[41]
	Multidrug-resistant *Escherichia coli*	Engineered endolysin derived from phage ΦEcSw	Engineering enhanced the ability of the endolysin to act against Gram-negative *E. coli*, demonstrating the feasibility of modifying phage enzymes to overcome the outer-membrane barrier.	Represents a proof-of-concept strategy; activity remains dependent on the engineered construct, bacterial strain, and experimental conditions.	[42]
	*Pseudomonas aeruginosa*	Lytic phage ZAM-Pa99; planktonic and biofilm assays	The phage demonstrated lytic activity against a substantial proportion of tested isolates and reduced established biofilms in vitro.	Supports antibiofilm potential but does not address in vivo penetration, immune clearance, or pharmacokinetic constraints.	[28]
	MDR *Klebsiella pneumoniae*	Phage cocktails alone and in combination with colistin or tigecycline	Phage combinations enhanced bacterial suppression, and phage–antibiotic combinations showed improved antibacterial activity under selected experimental conditions.	Synergy was dependent on the phage–antibiotic–strain combination and therefore should not be assumed to be generalizable across isolates or antimicrobial classes.	[29]
	Colistin-resistant *A. baumannii*	Phage selection combined with colistin exposure	Acquisition of phage resistance was associated with alterations in bacterial envelope architecture and, in the examined strain, increased susceptibility to colistin.	Illustrates a potentially exploitable evolutionary trade-off, but the finding was demonstrated in a restricted experimental system and requires validation across genetically diverse clinical isolates.	[34]
Preclinical: animal models	MDR *P. aeruginosa* sepsis in mice	Lytic phage PaeP_Ls administered in a murine sepsis model	Phage treatment reduced bacterial burden and inflammatory responses and improved survival relative to untreated infected animals.	Demonstrates in vivo activity in an acute infection model; translation to human infection remains uncertain because phage pharmacokinetics, immune interactions, and dosing differ substantially between experimental models and patients.	[43]
	MRSA experimental pneumonia in rats	Aerosolized and intravenous phage administration	Combined aerosolized and intravenous administration produced greater survival than either phage route alone, with survival reaching approximately 91% in the combined-treatment group.	Supports the importance of delivery route and local phage exposure, but results derive from a controlled experimental pneumonia model and cannot establish comparative clinical efficacy.	[44]
	CTX-M-15-producing *E. coli* ST131 sepsis and meningitis in neonatal rats	Strain-specific lytic bacteriophage	Phage administration improved outcomes in experimental sepsis and meningitis caused by the high-risk ST131 clone.	Provides proof of in vivo efficacy against a clinically important resistant lineage, although the narrow experimental setting and strain specificity limit generalization.	[45]
	MDR *K. pneumoniae* infection model	Phage cocktails combined with antibiotics	In addition to in vitro synergy, selected combinations reduced bacterial burden in an animal infection model.	Supports further investigation of phage–antibiotic combinations, although optimized combinations may need to be established individually for different strains and drugs.	[29]
Clinical evidence: compassionate-use and observational cohorts	Drug-resistant *Mycobacterium* infections	Personalized phage therapy in 20 patients treated on a compassionate-use basis; intravenous, aerosolized, or combined administration	Favorable clinical or microbiological responses were reported in 11 patients, and no adverse reactions were attributed to phage administration. Neutralizing antibodies developed in several intravenously treated patients and may have contributed to treatment failure in some cases.	One of the larger compassionate-use series, but treatment was individualized and frequently accompanied by antibiotics, surgery, or other interventions. The absence of a control group prevents attribution of clinical improvement specifically to phage therapy.	[37]
	Diverse persistent or refractory infections treated through the Israeli Phage Therapy Center	Personalized compassionate-use phage treatment; 20 treatment courses in 18 patients	Favorable clinical outcomes were reported in the majority of evaluable patients, although microbiological eradication was less consistent and treatment failures also occurred.	Provides real-world feasibility and safety information, but heterogeneous infections, individualized regimens, concomitant antibiotics, and absence of controls substantially limit efficacy inference.	[38]
	Refractory *P. aeruginosa* infections	PASA16-based personalized phage therapy, generally administered with antibiotics; compassionate-use case series	Favorable outcomes were reported in most treated patients, although treatment failures occurred and minor adverse effects were observed.	The uncontrolled design and concomitant antimicrobial treatment preclude determination of the independent contribution of phage therapy. These findings are therefore best regarded as signals supporting prospective evaluation.	[39]
	Selected severe infections, including CRAB pulmonary infection, CRAB/CRKP pulmonary coinfection, and chronic *S. aureus* prosthetic joint infection	Individualized nebulized, intravenous, and/or locally administered phage regimens	Individual reports described microbiological and/or clinical improvement in otherwise difficult-to-treat infections.	These cases illustrate feasibility and potential therapeutic activity but should not be interpreted as efficacy evidence because of their anecdotal nature, concurrent interventions, individualized treatment, and susceptibility to publication bias.	[47,48,50]
Prospective/interventional clinical studies	*S. aureus*-positive chronic rhinosinusitis	Intranasal AB-SA01 phage cocktail; open-label, first-in-human phase I study, *n* = 9	Intranasal phage administration was generally well tolerated, with no serious treatment-related safety signal. Reduction or eradication of *S. aureus* was observed in some participants.	The study was primarily designed to assess safety and tolerability. Its small sample size, open-label design, and absence of a control group do not permit conclusions regarding efficacy.	[52]
	Chronic nonhealing wounds infected with various bacteria	Personalized topical phage preparations; prospective uncontrolled study, *n* = 20	Clinical and microbiological improvement was reported following topical phage administration; complete healing by day 21 was documented in 7 patients, while other wounds showed granulation and improvement.	There was no vehicle-only or standard-care comparator; the cohort was small, and not all wounds were followed to complete resolution. Details of formulation and topical application were also limited. Consequently, the study provides a preliminary therapeutic signal but does not establish efficacy.	[53]
	Severe *S. aureus* infections, including infective endocarditis and septic shock	Intravenous AB-SA01 administered adjunctively to optimized antibiotic therapy; single-arm, non-comparative study, *n* = 13	Intravenous phage therapy was generally well tolerated, with no adverse reactions attributed to AB-SA01. Clinical improvement was observed in a number of patients.	The primary purpose was assessment of safety and tolerability. All patients received optimized antimicrobial therapy. There was no control arm, and the study was not designed to determine efficacy. Clinical outcomes therefore cannot be attributed independently to phage treatment.	[54]
	*P. aeruginosa*-infected burn wounds	PP1131 phage cocktail versus standard care; randomized, controlled, double-blind phase I/II PhagoBurn trial	Phage treatment was generally tolerated, but reduction in bacterial burden occurred more slowly in the phage group than with standard care. The administered phage preparation had undergone substantial loss of titre, resulting in exposure markedly below the intended dose.	PhagoBurn provides an important counterpoint to uncontrolled favorable reports. It demonstrates both the feasibility and the practical challenges of controlled phage trials, particularly product stability, dose standardization, and manufacturing consistency; it did not demonstrate superiority over standard care.	[56]
Clinical implementation	Diverse refractory or difficult-to-treat bacterial infections	PHAGEinLYON multidisciplinary clinical pathway	A structured pathway was established to coordinate patient selection, phage sourcing, microbiological testing, regulatory processes, administration, and clinical follow-up.	Demonstrates the feasibility of integrating personalized phage access into a hospital framework, but implementation experience should not be interpreted as evidence of therapeutic efficacy.	[55]
Evidence synthesis	Difficult-to-treat bacterial infections across multiple pathogens and clinical syndromes	Systematic review of clinical phage-therapy experience	Available clinical reports generally indicated acceptable tolerability and reported favorable outcomes in many patients.	The evidence base was dominated by heterogeneous compassionate-use cases and uncontrolled studies, with substantial variation in phage products, dosing, routes, concomitant antibiotics, and outcome definitions. Consequently, the available literature supports continued clinical investigation but remains insufficient for robust estimates of comparative efficacy.	[46]

## Data Availability

No new data were created or analyzed in this study. Data sharing is not applicable to this article.

## Abbreviations

The following abbreviations are used in this manuscript:

AMR	Antimicrobial resistance
MDR	Multidrug-resistant
XDR	Extensively drug-resistant
PDR	Pandrug-resistant
CRE	Carbapenem-resistant Enterobacterales
MRSA	Methicillin-resistant *Staphylococcus aureus*
CRAB	Carbapenem-resistant *Acinetobacter baumannii*
dsDNA	Double-stranded DNA
ssDNA	Single-stranded DNA
dsRNA	Double-stranded RNA
ssRNA	Single-stranded RNA
RBPs	Receptor-binding proteins
R–M	Restriction–modification
PAS	Phage–antibiotic synergy
PK/PD	Pharmacokinetics and pharmacodynamics
GMP	Good Manufacturing Practice
PMDA	Pharmaceuticals and Medical Devices Agency
COPD	Chronic obstructive pulmonary disease
ESBL	Extended-spectrum β-lactamase
